# Supplementary figures and images for: Species-wide survey of the expressivity and complexity spectrum of traits in yeast
Source: PLoS Genet. 2024 Jan 18;20(1):e1011119. doi: 10.1371/journal.pgen.1011119 (PMC10826966; doi:10.1371/journal.pgen.1011119)

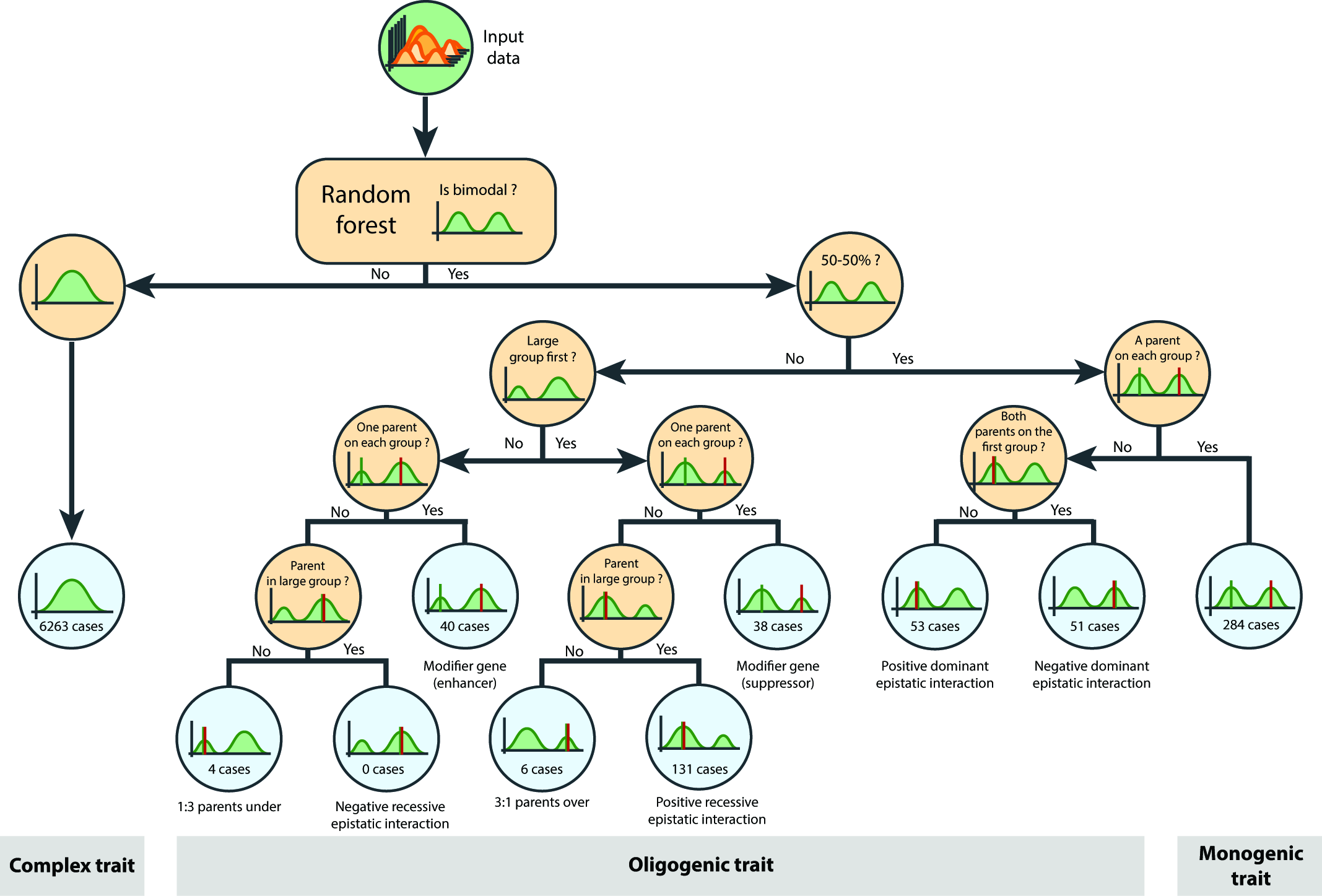

Supplement: S1 Fig — Results of the random forest, the proportions of the groups of spores and the phenotypes of the parents were used as the input for a decision tree that classifies the phenotypic distribution in one of 8 types of genetic complexity that were then merged into 3 complexity levels (monogenic, oligogenic and complex). (TIF) [file pgen.1011119.s001.tif]

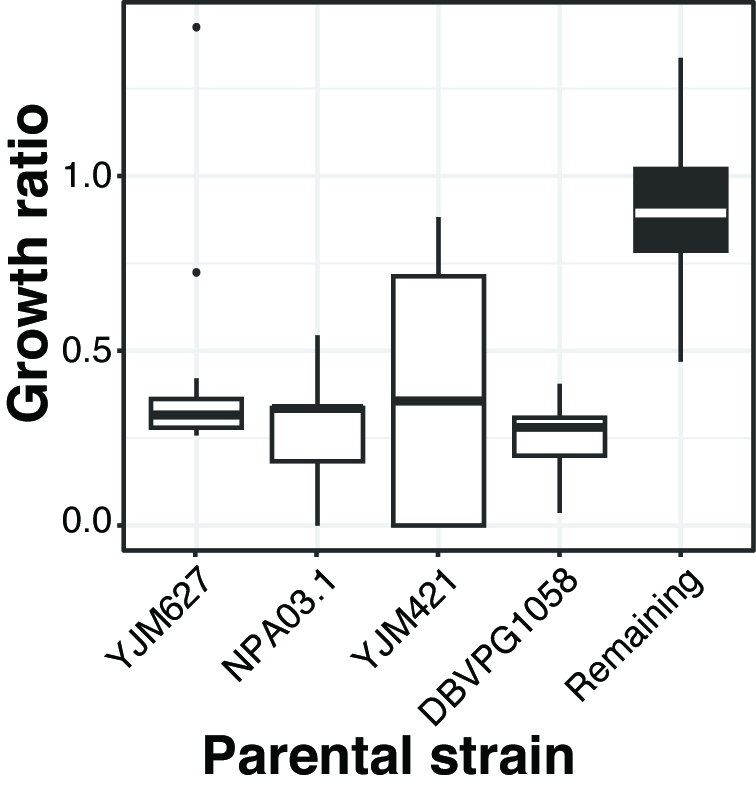

Supplement: S2 Fig — Comparison between the growth ratios of the 4 parental strains producing many monogenic and oligogenic cases and the 16 remaining parental strains. (TIF) [file pgen.1011119.s002.tif]

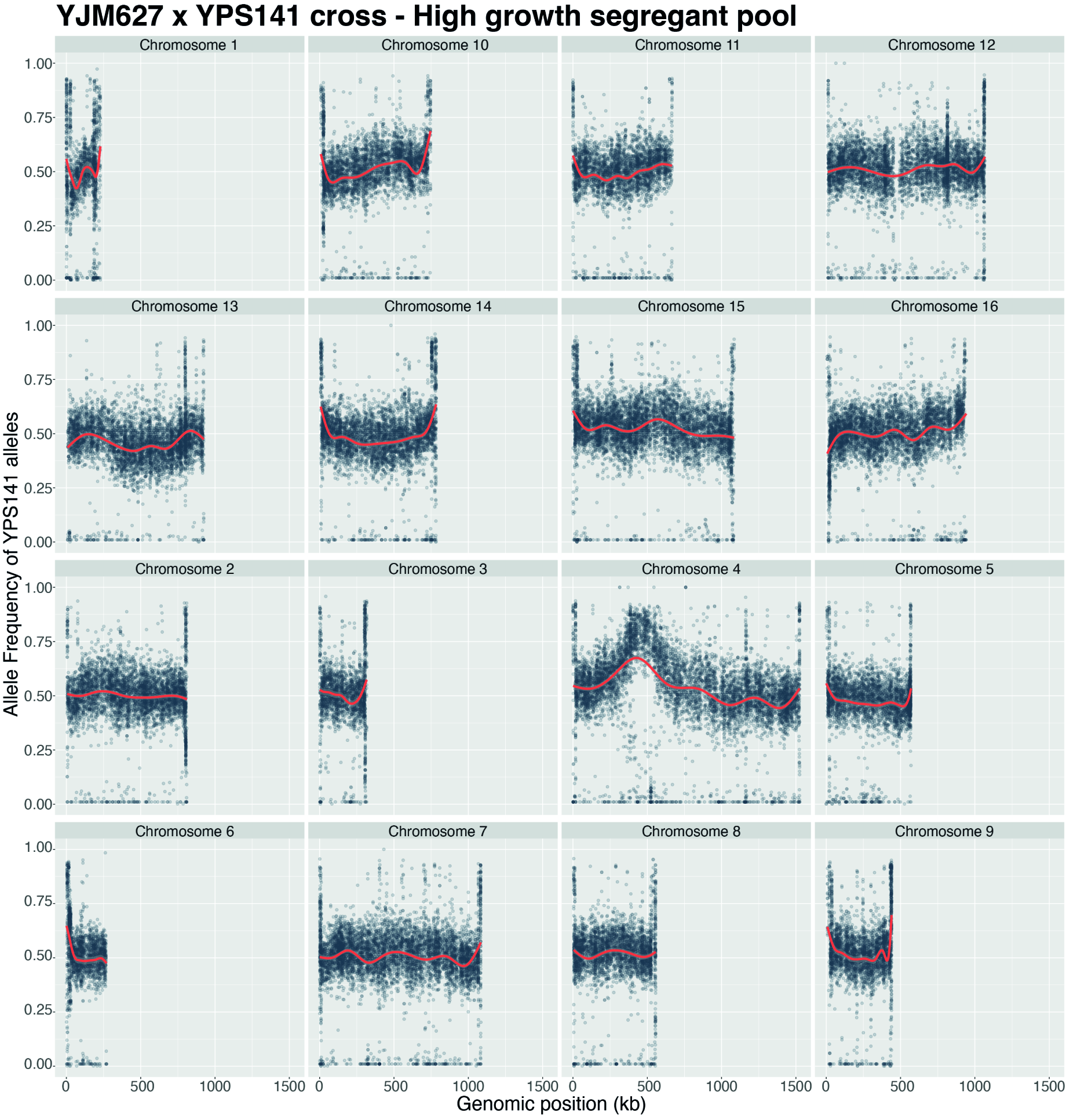

Supplement: S3 Fig — Frequency of the YPS141 alleles in the sequencing reads of the segregants with high growth on galactose 2%. Important deviation towards the YPS141 alleles is observed in chromosome 4 (400,000–500,000). (TIF) [file pgen.1011119.s003.tif]

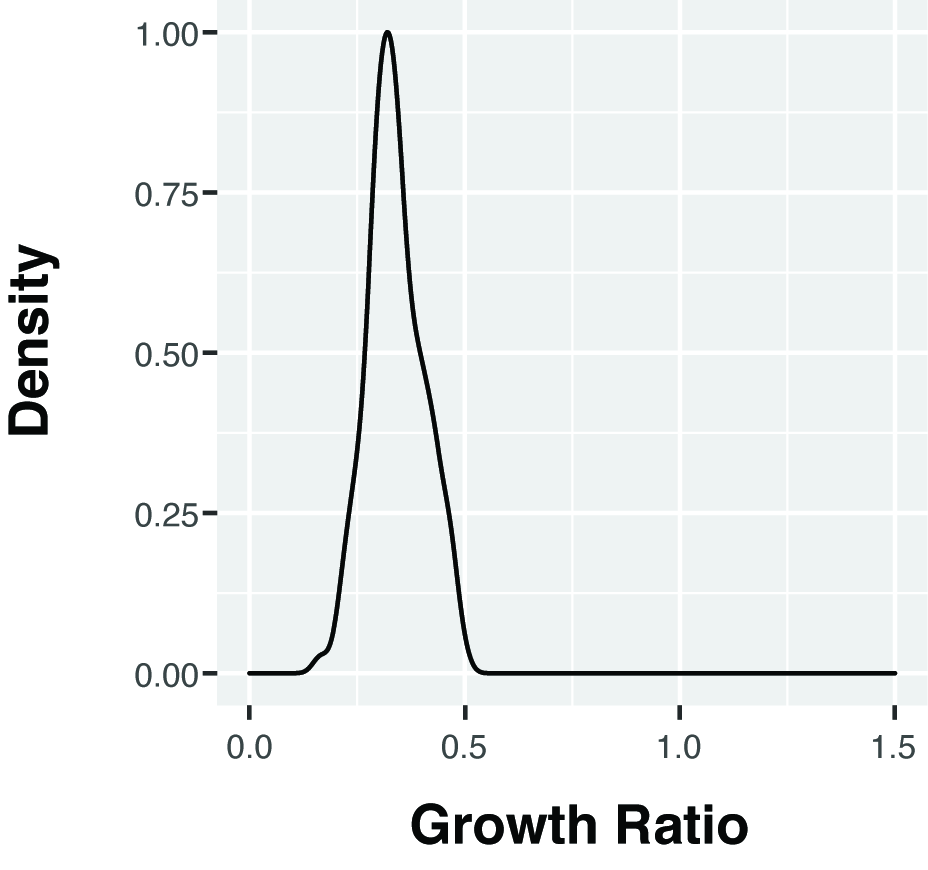

Supplement: S4 Fig — Distribution of the growth phenotypes on galactose 2% of the segregants from the cross of YJM627 and NPA03.1. All segregants display low growth indicating that the large effect loci in the two parents are under genetic linkage and therefore positioned in the same region. (TIF) [file pgen.1011119.s004.tif]

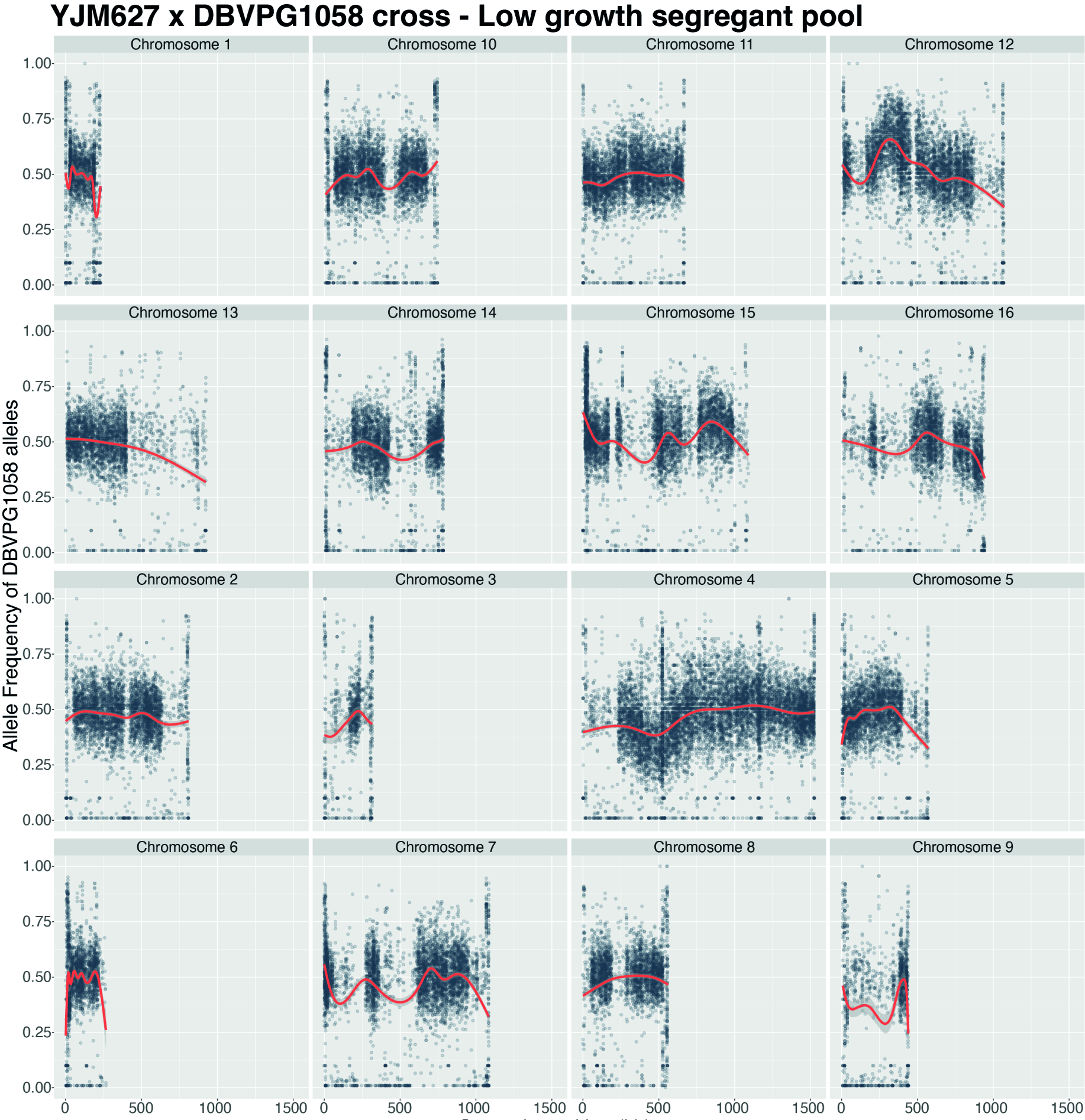

Supplement: S5 Fig — Frequency of DBVPG1058 alleles in the sequencing reads of the segregants with low growth on galactose 2%. Important deviations are observed in two regions. On chromosome 4, there is a decrease of DBVPG1058 allele frequency, suggesting a deviation toward YJM627. On chromosome 12, there is a significant increase of the DBVPG1058 allele frequency. (TIF) [file pgen.1011119.s005.tif]

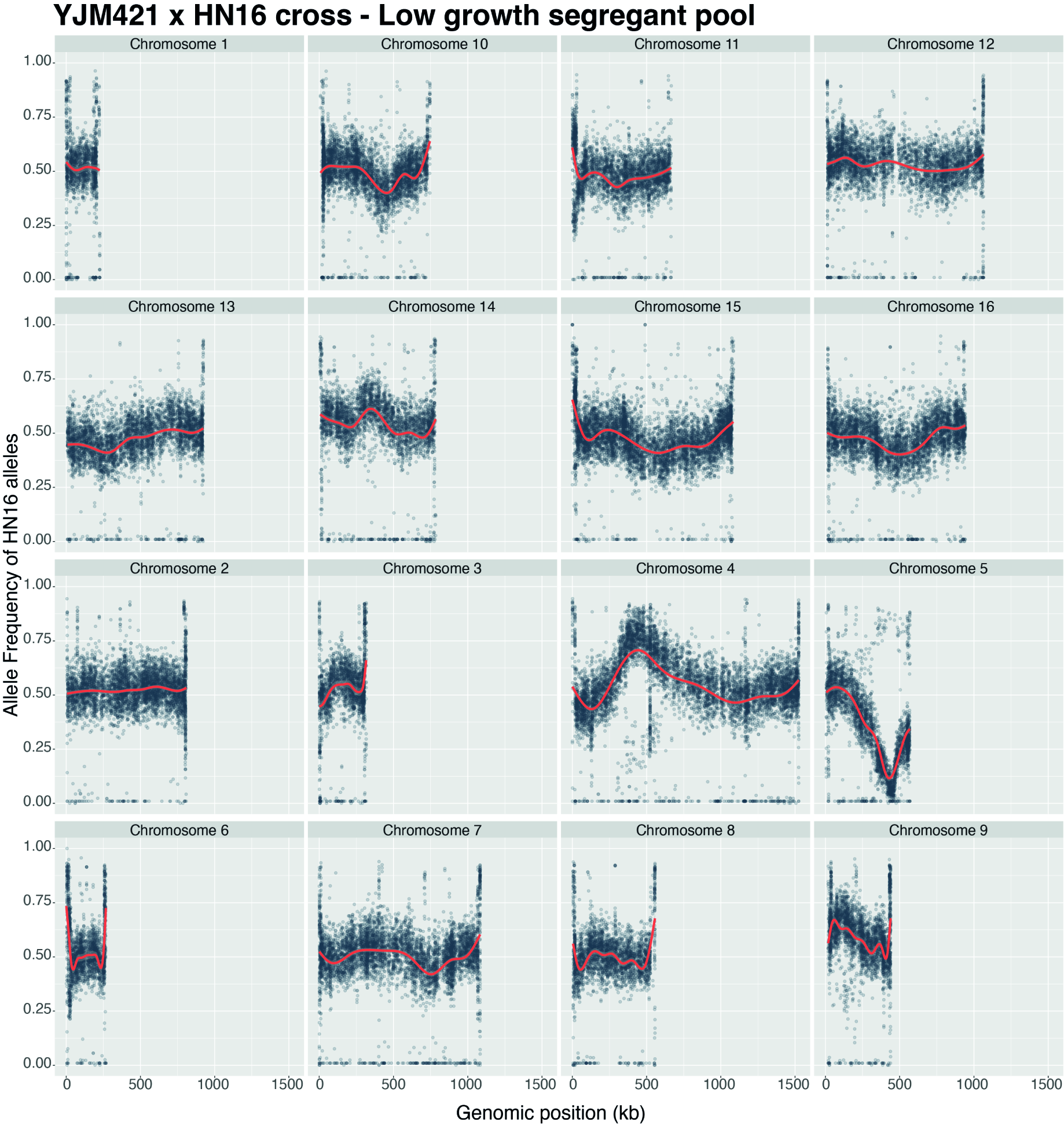

Supplement: S6 Fig — Two regions, on chromosome 4 (350,000–500,000) and chromosome 5 (400,000–500,000), show important deviations towards the HN16 and YJM421 alleles, respectively. (TIF) [file pgen.1011119.s006.tif]

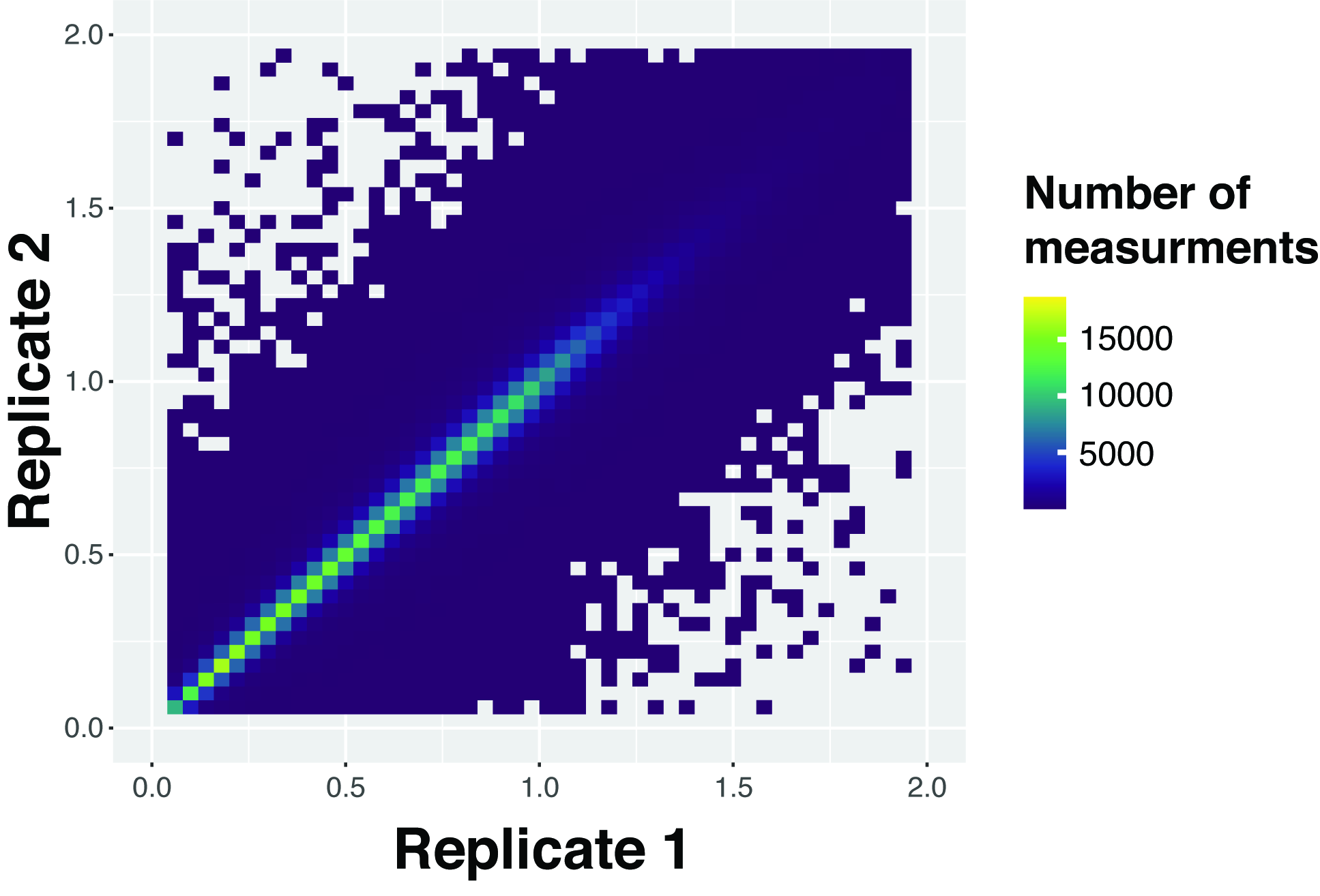

Supplement: S7 Fig — Correlation density between replicates of the same segregant on the same condition (R = 0.96, p-val< 2.2e-16). (TIF) [file pgen.1011119.s007.tif]
